# 

**Published:** 1858-03

**Authors:** 


### CONTENTS.

ORIGINAL ESSAYS AND COMMUNICATIONS.

Filling Teeth, By J. Taft, .......... 233
Valedictory Address, By C. B. Chapman, .......... 237
Professional Personalities, .......... 247
Remarks upon the Teeth of the Natives of India, .......... 251
A Separating Forcep, Valuable for Extracting Dens Sapientae, .......... 253
The Poverty of Dentists, .......... 257
"Novice" Noticed, .......... 260
The Ethics of the Dental Profession, By B. P. Aydelott, D.D., .......... 265
Report on Dental Progress, .......... 282
American Dental Convention, .......... 347
Improved Plates, By Dr. J. W. Wintet, .......... 348

PROCEEDINGS OF SOCIETIES.

Minutes and Discussions of the New York Dental Association, .......... 292
Minutes of the Ohio Dental College Association, .......... 316
Fourteenth Annual Meeting of the Miss. Valley Asso. of Dental Surgeons,—Minutes .......... 319
Fourteenth Annnal Meeting of the Miss. Velley Asso. of Dental Surgeons—Discussions .......... 326
Miscellaneous .......... 349
Editorial .......... 355

